# A green fluorescent protein-expressing murine tumour but not its wild-type counterpart is cured by photodynamic therapy

**DOI:** 10.1038/sj.bjc.6602953

**Published:** 2006-01-17

**Authors:** A P Castano, Q Liu, M R Hamblin

**Affiliations:** 1 BAR414, Wellman Center for Photomedicine, Massachusetts General Hospital, 40 Blossom Street, Boston, MA 02114, USA; 2 Department of Dermatology, Harvard Medical School, Boston, MA, USA; 3 Harvard-MIT Division of Health Sciences and Technology, Cambridge, MA, USA

**Keywords:** photodynamic therapy, green fluorescent protein, antitumour immunity, benzoporphyrin derivative, radiation-induced fibrosarcoma, fluorescence imaging

## Abstract

The ideal cancer treatment should both destroy the primary tumour and at the same time educate the immune system to recognise the tumour as foreign so that distant metastases will also be eradicated. Photodynamic therapy (PDT) involves the i.v. administration of photosensitisers followed by illumination of the tumour with red light producing reactive oxygen species that eventually cause vascular shutdown and tumour cell death by apoptosis and necrosis. Anti-tumour immunity is stimulated after PDT due to the acute inflammatory response, generation of tumour-specific antigens, and induction of heat-shock proteins. Green fluorescent protein (GFP) is used as an optical reporter to noninvasively image the progression of mouse tumours, and in addition, may act as a foreign (jellyfish) antigen. We asked whether GFP-expressing tumours could be used to monitor the response of tumour-bearing mice to PDT, and whether the tumour response differed when a nonimmunogenic tumour cell line was transduced with GFP. We injected RIF-1 or RIF-1 EGFP (stably transduced with a retroviral vector) cells in the leg of C3H/HeN mice and both the cells and tumour grew equally well. We used PDT with benzoporphyrin derivative and a short drug-light interval. There were complete cures and 100% mouse survival of RIF-1 EGFP while RIF-1 wild-type tumours all recurred. Cured mice were resistant to rechallenge with RIF-1 EGFP cells and a rechallenge with wild-type RIF-1 cells grew significantly slower. There was also slower RIF-1 EGFP rechallenge growth but no rejection when RIF-1 EGFP tumours were surgically removed. There was a low rate of PDT cure of tumours when RIF-1 cells were transduced with an empty retroviral vector. The presence of antibodies against EGFP in mouse serum suggests EGFP can act as a foreign antigen and PDT can then stimulate a long-term memory immune response.

It was estimated that approximately 563 700 Americans would die of cancer in 2004 corresponding to over 1500 deaths per day (Smith *et al*, 2004). Despite advances in early detection and treatment, most patients still cannot be treated effectively and eventually die, largely due to metastatic disease. The ideal cancer treatment should destroy both the primary tumour and at the same time educate the immune system to recognise the tumour as foreign so that, after the primary tumour is destroyed, distant metastases will also be eradicated.

Photodynamic therapy (PDT) uses a nontoxic photoactivatable dye or photosensitiser (PS) in combination with harmless visible light that produces reactive oxygen species and destroys tumour tissue (Dolmans *et al*, 2003). Photodynamic therapy is approved for multiple indications in the United States, and many other countries (Dougherty, 2002). Mechanisms that have been shown to be involved in the tumouricidal effect include direct cytotoxicity to tumour cells, shutting down of the tumour vasculature starving the tumour of oxygen or nutrients, and the induction of a host immune response (Dougherty *et al*, 1998). The precise mechanisms involved in the PDT-mediated induction of antitumour immunity are not yet completely understood (Korbelik, 1996). Among the potential contributing factors are alterations in the tumour microenvironment via stimulation of proinflammatory cytokines and direct effects of PDT on the tumour that increase immunogenicity (Canti *et al*, 2002). In contrast to common cancer therapies such as surgery, radiation therapy and chemotherapy that are all more or less immunosuppressive, PDT can stimulate the immune response to cancer.

Green fluorescent protein (GFP) was originally isolated from the jellyfish, *Aequorea victoria* (Chalfie, 1995). Enhanced GFP (EGFP) is a red shifted variant, which fluoresces much more intensely than wild-type GFP (Sacchetti *et al*, 2000). Hoffman and coworkers (Hoffman, 1999a, 1999b, 2001, 2002a) have introduced the concept of *in vivo* monitoring of GFP expressing tumours using macroscale fluorescence imaging, There are some reports that GFP can be immunogenic when expressed in mouse tumours (Stripecke *et al*, 1999; Gambotto *et al*, 2000; Brown *et al*, 2001). In this report, we asked whether GFP-expressing tumours could be used to monitor the response of tumour-bearing mice to PDT, and whether the tumour response differed when a nonimmunogenic tumour cell line was transduced with GFP.

## MATERIALS AND METHODS

### Cell line and tissue culture conditions

The radiation-induced fibrosarcoma (RIF-1) tumour cells originally characterised by Twentyman *et al* (1980), were grown in RPMI 1640 media containing HEPES, glutamine, 10% fetal calf serum, 100 U ml^−1^ penicillin and 100 *μ*g ml^−1^ streptomycin. They were collected for injection by washing with PBS without Ca^2+^ and Mg^2+^, and adding trypsin-EDTA to the plate for 10 min at 37°C.

### Stable expression of EGFP

The retroviral plasmid vector (pLEGFP-N1, cat# 6059-1) expressing the enhanced GFP (EGFP) was purchased from BD Clontech (Palo Alto, CA, USA), and propagated in *Escherichia coli* DH5a (Sigma, St Louis, MO, USA). The plasmid was then purified with the Plasmid Kit from Qiagen (Valencia, CA, USA) as instructed. A packaging cell line (AmphoPack-293, BD Clontech) was transfected with the plasmid vector using Lipofectamine 2000 (Invitrogen, Carlsbad, CA, USA) as described for the reagent. Medium containing the retrovirus was collected after 48 h post-transfection, filtered through a 0.45 *μ*m membrane, and used to infect the RIF-1 cells, as described in the retroviral gene expression user manual. Radiation-induced fibrosarcoma cells permanently expressing EGFP (RIF-1 EGFP) were initially cultured in 2 mg ml^−1^ G418 and selected for EGFP expression by limiting dilution and fluorescence microscopy. This was carried out in order to pick a stable and bright level of fluorescence. Cells were not routinely maintained with G418 but were used for a maximum of six passages and EGFP expression levels were checked at passage 6 by FACS analysis. Additional RIF-1 cells were transduced with the empty retroviral vector (pLNCX2, cat# 6102-1) lacking the gene for EGFP and selected and routinely cultured in the presence of 2 mg ml^−1^ G418. These were termed RIF-1 EPV.

### Mice and tumour model

All animal experiments were approved by the Subcommittee on Research Animal Care of Massachusetts General Hospital and were in compliance with NIH guidelines. Male C3H/HeN mice (Charles River Labs, Boston, MA, USA), weighing 20–25 g were shaved on the right thigh and depilated with Nair (Carter-Wallace Inc New York, NY, USA). Mice were anaesthetised with an i.p. injection of ketamine/xylazine cocktail (90 mg kg^−1^ ketamine, 10 mg kg^−1^ xylazine). One million RIF-1 cells or RIF-1 EGFP cells were injected subcutaneously in one mid-thigh area suspended in 100 *μ*l PBS. Tumours grew predictably in all mice and reached a size of 5–6-mm diameter 8–9 days after injection at which time they were used for PDT. Some mice had their tumours removed by surgical amputation of the tumour bearing leg at the same time point (5–6 mm diameter).

### Anti-EGFP serum antibodies

An ELISA assay for mouse anti-EGFP detection was modified from that of Brown *et al* (2001). Briefly, recombinant EGFP (BD Clontech) was diluted into 10 mM Tris pH 8.5 at 2 *μ*g ml^−1^, and 50 *μ*l was added into each well of 96-well MaxiSorp ELISA plate (Nunc, Rochester, NY, USA), incubated at room temperature for 90 min. The wells were then blocked by incubating with PBS containing 1% BSA for 1 h at room temperature, and washed three times with PBS containing 0.05% Tween 20 (PBS-T). Mouse serum (10 *μ*l) was diluted into 100 *μ*l PBS-T and added to the wells, incubated for 2 h at room temperature. Then the serum was removed and the wells washed three times with the PBS-T. Captured mouse anti-EGFP was detected with an alkaline phosphatase-conjugated secondary antibody (Biostain, Foster City, CA, USA) and D-nitrophenylphosphate (Sigma). Optical absorbance at 405 nm was read at 60 min on a plate reader (Molecular Dynamics). A calibration curve was constructed using known amounts of mouse IgG coated on wells of a plate and the same alkaline phosphatase-conjugated secondary antibody, that allowed the OD at 405 nm to be converted to *μ*g ml^−1^ IgG.

### *In vivo* fluorescence imaging

Mice bearing GFP expressing tumours were lightly anaesthetised with an i.p. injection of ketamine/xylazine cocktail (45 mg kg^−1^ ketamine, 5 mg kg^−1^ xylazine) and were imaged in a macrofluorescence imaging system (Lightools Research, Encinatas, CA, USA). Incandescent white light (Illumatools, Lightsource Research) was filtered through a 450–490 band pass filter and a liquid light guide to provide illumination from two parallel angled light diffusers positioned 25 cm above the mouse. Emission light passed through a 500-nm long pass filter into an Optronics CE (Goleta, CA, USA) colour charge-coupled device camera via a f1.4 zoom lens. The camera was controlled by Magnafire SP software (Optronics) and an acquisition time of 2.468 s and a white balance of 2800 K were used to capture images of 1280 by 1024 pixels. Adobe Photoshop (San Jose, CA, USA) was used to compile time course composite images.

On day 20 after tumour injection tumour-bearing animals were killed with carbon dioxide, and dissected with a midline skin incision extending from the caudal abdomen to the lower jaw, the skin was reflected and an incision into the abdominal cavity just cranial to the external genitalia permitted visualisation of the viscera. The incision was extended to the rib cage by cutting abdominal musculature on both sides and reflected over the thorax to expose the abdominal contents; the liver was reflected cranially to expose the stomach. The spleen and the lymph nodes in the retroperitoneum were seen. All the necropsy procedures were followed with GFP imaging to detect any metastasis.

### Photodynamic therapy

BPD (liposomal benzoporphyrin derivative mono-acid ring A, Verteporfin for Injection) was a generous gift from QLT Inc. (Vancouver, BC, Canada) as a lyophilised powder, which was reconstituted with 5% dextrose solution and injected at a dose of 2 mg kg^−1^ in 0.1 ml solution via the lateral tail vein. Photodynamic therapy was carried out at 15 min after injection for BPD using an 1 W solid state diode laser (High Power Devices Inc., North Brunswick, NJ, USA) emitting light at 690 nm (±2 nm). The laser was coupled into a 400-*μ*m fibre via a SMA connector and light from the distal end of the fibre was focused into a uniform spot with an objective lens (No 774317, Olympus, Tokyo, Japan). The spot had a diameter of 1.2 cm and was positioned so that the entire tumour and a surrounding 2–3 mm of normal tissue was exposed to light. Mice were anaesthetised as described above and the tumour bearing leg positioned under the spot. A total fluence of 150 J cm^−2^ was delivered at a fluence rate of 100 mW cm^−2^. Some mice had their RIF-1 or RIF-1 EGFP tumours illuminated without having received a prior injection of BPD. At the completion of the illumination mice were allowed to recover in an animal warmer until they resumed their normal activity.

### Follow-up of mice and tumour rechallenge

Mice were examined three times a week. They were weighed and the orthogonal tumour dimensions (*a* and *b*) were measured with vernier calipers. The tumour volume was calculated according to the formula, volume=4/3 *π*{(*a*+*b*)/4}^3^. Tumours were treated when 5–6 mm in diameter (volume ≈70–110 mm^3^). Mice were imaged in the GFP camera if their tumour was EGFP expressing. Mice were killed according to the protocol when the tumours reached a largest diameter of 1 cm. Mice were considered cured when the tumour did not return after 60 days. Cured mice or surgically amputated mice were rechallenged with one million tumour cells (RIF-1 EGFP or RIF-1) injected in the opposite leg to the one that had previously borne the primary tumour.

### Statistics

All values are expressed as±s.e. of the mean. Comparison between two means was carried out using the Mann–Whitney *U*-test. Survival analysis was performed using the Kaplan–Meier method. Survival curves were compared, and differences in survival were tested for significance using a log-rank test in the computer program GraphPad Prism (GraphPad Software Inc., San Diego, CA, USA). The tumour growth curves were analysed by transforming the data to a logarithmic scale and comparing the slopes. *P*-values of <0.05 were considered significant.

## RESULTS

### Growth rate of RIF-1 EGFP cells and tumours

**Figure 1:**
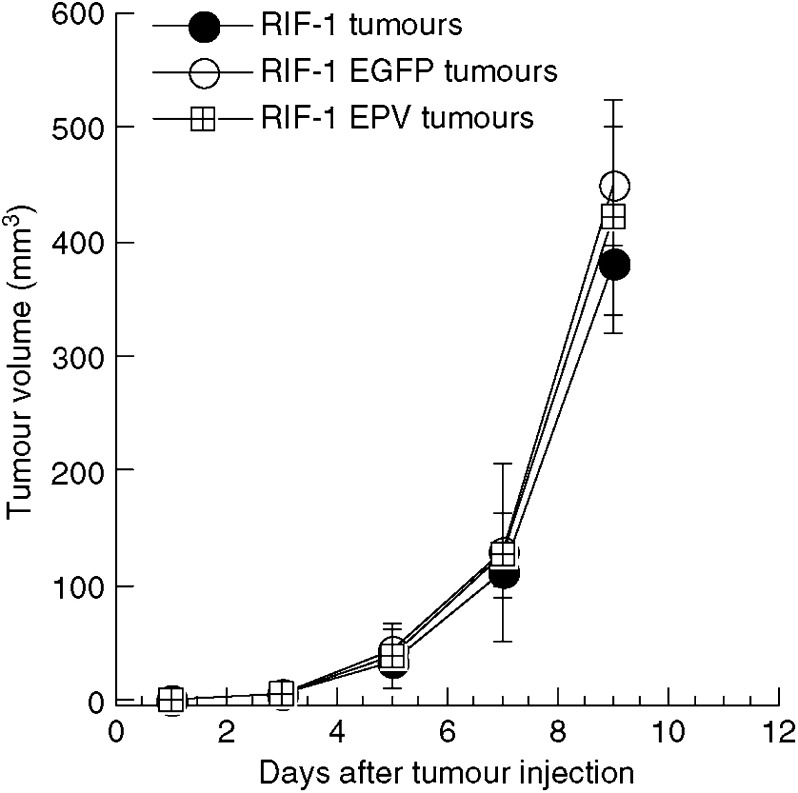
Growth rate of RIF-1, RIF-1 EGFP and RIF-1 EPV tumours injected subcutaneously in C3H/HeN mice.

It was necessary to ensure that the stable expression of the EGFP gene via a retrovirus, did not have any deleterious effects on either the growth rate of the tumour cells in tissue culture, or the growth rate of the tumours when subcutaneously implanted into C3H mice. The doubling times of the RIF-1, RIF-1 EGFP and RIF-1 EPV cells in tissue culture were not statistically different (data not shown). As can be seen from Figure 1 there were no significant differences in the growth of the three tumours.

### Fluorescence imaging of growth of RIF-1 EGFP tumour

**Figure 2:**
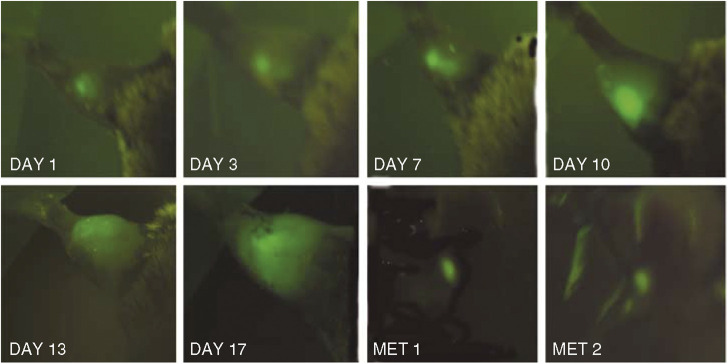
Series of *in vivo* fluorescence images of a RIF-1 EGFP tumour growing in the leg of a C3H mouse. The images labeled MET 1 and 2 were captured at autopsy and show metastases in the liver.

Fluorescence imaging was used to noninvasively visualise the progression of the EGFP-expressing tumours. In Figure 2 we show the progression of an untreated RIF-1 EGFP tumour that was permitted to grow until the 17th day (tumour diameter 12 mm). The mouse was killed and the necropsy procedure carried out as described. Using the macrofluorescence imaging system, we found two liver metastases with sizes between 2 and 3 mm, without any other organ compromised, and we did not find any lymph node metastasis.

### Anti-EGFP serum antibodies

**Figure 3:**
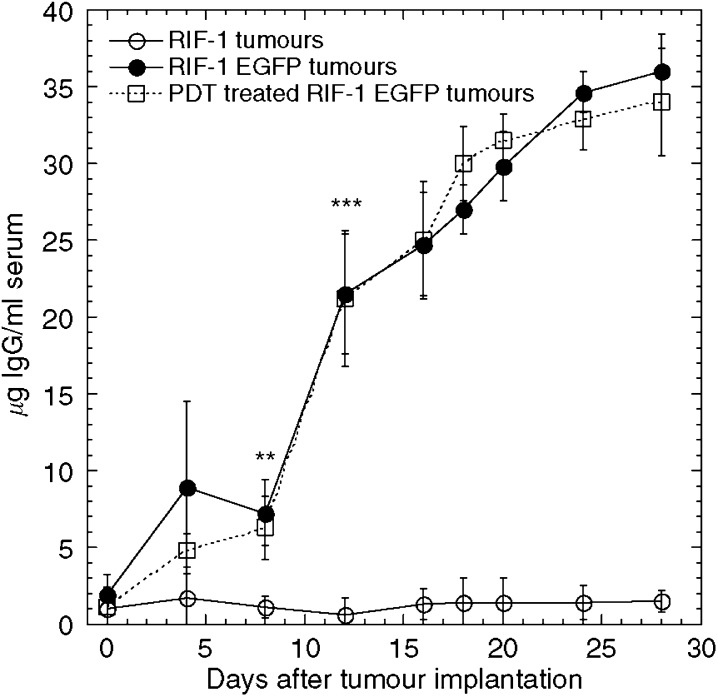
Detection of anti-EGFP antibodies. Recombinant EGFP was coated to the surface of 96-well ELISA plate, and serum was added and incubated as described in Materials and Methods. Anti-EGFP antibody in serum captured was detected with alkaline phosphatase conjugated-secondary antibody and chromogenic substrate D-nitrophenylphosphate. OD405 was read at 60 min. Data represent the averages of triplicates and two independent experiments. Significantly different from wild-typeRIF-1: ^**^P<0.01; ^***^P<0.001 by Mann–Whitney *U*-test.

Blood (100 *μ*l) was withdrawn via the orbital plexus from mice bearing RIF-1 or RIF-1 EGFP tumours, allowed to clot and serum obtained. Serum samples were analysed for the presence of anti-EGFP antibodies as described in Materials and Methods. Figure 3 shows that mice with RIF-1 EGFP but not RIF-1 tumours developed antibodies to EGFP by the 12th day after tumour injection. We also asked whether PDT of RIF-1 EGFP tumour bearing mice led to a drop in their titre of anti-EGFP antibodies and as can be seen there was no difference between the control and PDT-treated mice.

### PDT response

**Figure 4:**
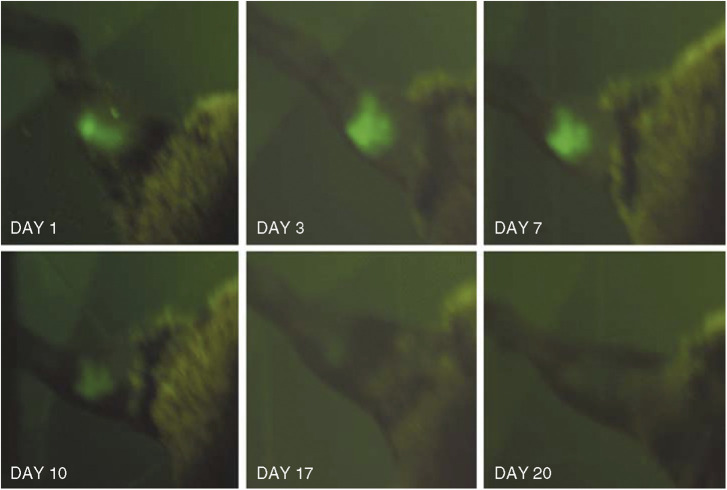
Series of *in vivo* fluorescence images of a RIF-1 EGFP tumour growing in the leg of a C3H mouse. The mouse was treated with BPD-mediated PDT on day 9.

**Figure 5:**
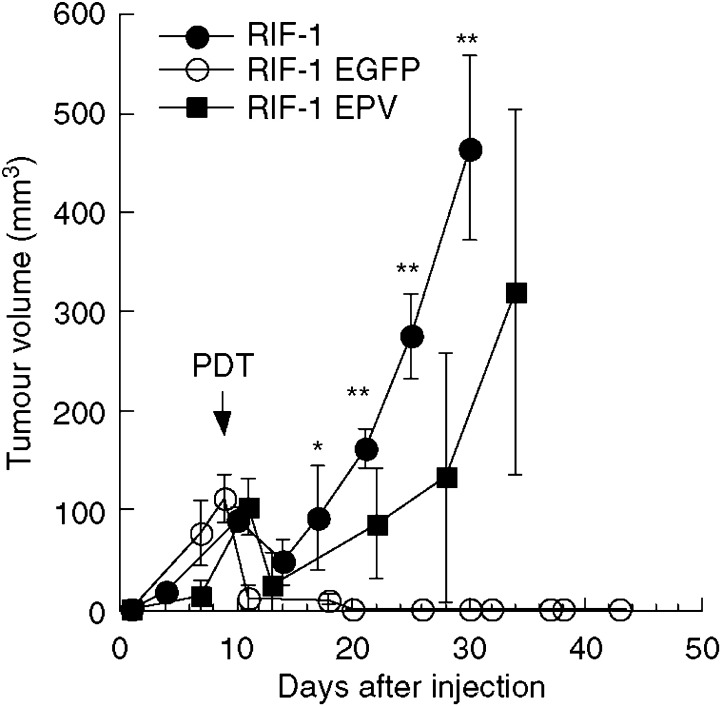
Tumour response of wild-type RIF-1, RIF-1 EGFP and RIF-1 EPV tumours that had been treated with BPD-mediated PDT as described. Tumour volume was calculated from dimensions measured with vernier calipers twice a week. There were 10 mice in RIF-1, 13 mice in RIF-1 EGFP and 12 mice in RIF-1 EPV groups, and bars are s.e.m. Significant differences between RIF1 and RIF1 EGFP; ^*^P<0.05 and ^**^P<0.001 by Mann–Whitney *U*-test.

Wild-type RIF-1 tumours were treated with PDT at days 8–9 (when tumours had a diameter of 5–6 mm). We used BPD (2 mg kg^−1^ i.v. followed after 15 min by 150 J cm^−2^ delivered at 100 mW cm^−2^). At 1 to 2 days after PDT the mice exhibited a black eschar confined to the area formerly occupied by the tumour, It was difficult to determine how much tumour was left under the black eschar but in all mice the volume of the tissue measured decreased after PDT. However, in all mice the tumours regrew, frequently forming a ring of viable rapidly growing tumour outside the black eschar. When RIF-1 EGFP tumours were treated under the same conditions with BPD-PDT we observed interesting and surprising differences in the responses. Firstly the tumour shrinkage was more marked with RIF-1 EGFP tumours than was found with RIF-1 tumours. Secondly, the subsequent behavior of the tumours was very different. The RIF-1 EGFP tumours disappeared completely by day 20 and did not return during the 40 days they were followed. The progress of one mouse bearing a RIF-1 EGFP tumour treated with BPD-PDT and monitored with fluorescence imaging is depicted in Figure 4. The reduction in EGFP fluorescence is clearly visible 1 day after PDT, and subsequent imaging at later time points revealed no remaining fluorescence. The differences in mean tumour size between the wild-type and EGFP-transduced tumours when treated with BPD-PDT is shown in Figure 5. There was no difference in growth rate between RIF-1 tumours or RIF-1 EGFP tumours exposed to red light without BPD and the growth rate of untreated RIF-1 or RIF-1 EGFP tumours (data not shown).

**Figure 6:**
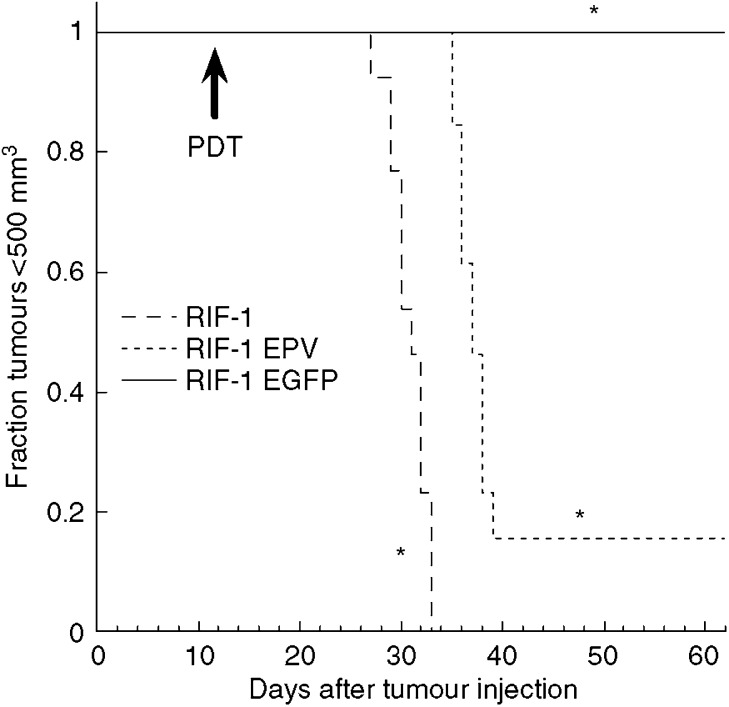
Kaplan–Meier curve. Mice bearing RIF-1, RIF-1 EGFP and RIF-1 EPV tumours were treated with BPD-PDT as described. Mice were killed when tumours reached a diameter of 1 cm. ^*^All three curves were significantly different (*P*<0.01) by log-rank analysis.

In order to check whether the retrovirus used to produce stable expression of EGFP was involved in this altered response to PDT, we produced RIF-1 cells that had been infected with a retrovirus produced from an empty plasmid vector (RIF-1 EPV). There was no difference between *in vivo* tumour growth rates observed in mice bearing these RIF-1 EPV tumours and either of the other RIF-1 variants tested previously (Figure 1). When mice bearing RIF-1 EPV tumours were treated with BPD-PDT there were two out of 12 mice that had long-lasting responses, that is, no local recurrence of tumour in 60 days. The remaining 10 out of 12 demonstrated local recurrence but these recurrent tumours grew slower than the recurrent wild-type RIF-1 tumours as shown in Figure 5, although the error bars are large due to combining cured and tumour progressing animals. The Kaplan–Meier survival curves (time to kill due to tumour dimensions exceeding 1 cm) for the three groups of tumours are presented in Figure 6.

**Figure 7:**
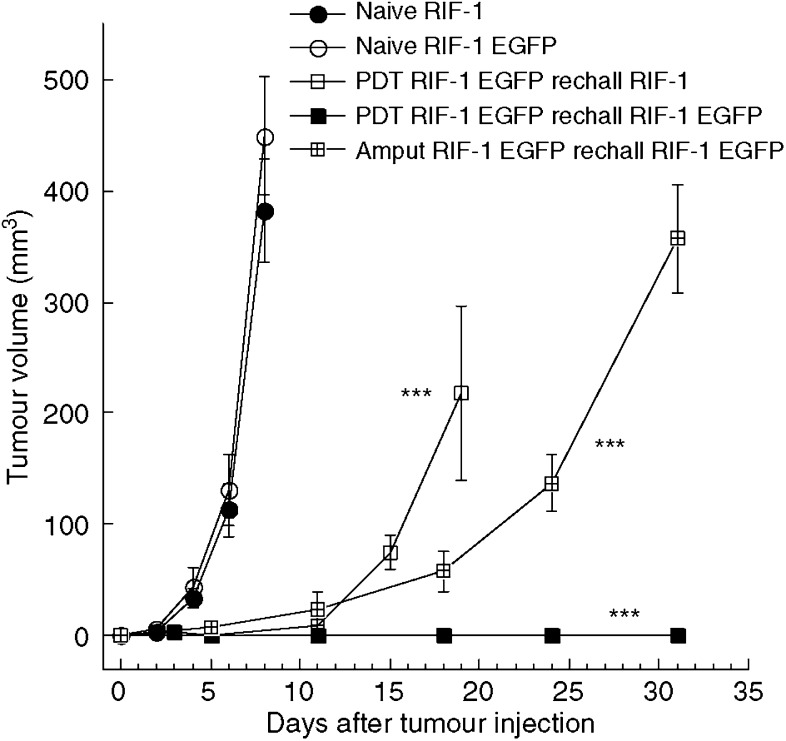
Tumour growth curves of naïve mice or cured mice rechallenged with tumour cells. Mice that had been cured of RIF-1 EGFP tumours by PDT or surgery were rechallenged with RIF-1 EGFP or wild-typeRIF-1. Tumour volume was calculated from dimensions measured with vernier calipers twice a week. There were five mice in each group. ^***^The curves showed significant differences compared to naïve mice (*P*<0.001) in tumour growth rate by logarithmic transformation and slope comparison.

Mice that were cured of RIF-1 EGFP tumours by BPD-PDT, mice that had their RIF-1 EGFP tumours surgically removed and untreated control mice were rechallenged by a second injection of 1 million RIF-1 EGFP cells after they had been tumour-free for 60 days. All the mice that had been cured of RIF-1 EGFP tumours by BPD-PDT rejected the subsequent tumour rechallenge (0 out of eight grew tumours), while all of the surgically cured mice (five out of five), and all of the naïve mice that were challenged with RIF-1 EGFP cells grew tumours (five out of five). However, the growth rate of the surgically cured mice rechallenged with RIF-1 EGFP was significantly slower than the growth rate of naive RIF-1 EGFP tumours (*P*<0.001 by comparing slopes of logarithmic transformed data). Five mice that had been cured of RIF-1 EGFP tumours and rejected a subsequent rechallenge with RIF-1 EGFP tumour cells were rechallenged with one million wild-type RIF-1 cells together with naïve mice. The RIF-1 tumours grew in all the mice but again the rate of growth of RIF-1 cells in mice cured of RIF-1 EGFP tumours by PDT was significantly slower than the rate in naïve mice (*P*<0.001 by comparing slopes of logarithmic transformed data) than in naïve mice (Figure 7). There were no long-term survivors in this group of mice.

## DISCUSSION

In this study, we have shown that there are significant and unexpected differences in the response to PDT of mouse tumours that are either wild-type or stably expressing EGFP. The repetitive imaging of mice with GFP-expressing tumours using a macrofluorescence imaging system is very useful tool to follow tumour progression and to test new therapeutic approaches (Kamiyama *et al*, 2002; Wang *et al*, 2003). However, many of these reports on GFP-tumour imaging used human tumour cell lines expressing GFP and therefore were constrained to employ immunosuppressed nude or SCID mice (Yamamoto *et al*, 2003). This fact may have led to a delay in the realisation that the jellyfish protein GFP can act as a foreign model tumour antigen in immunocompetent mice. Similar sea-borne materials and their derivatives, such as glycated chitosan, have also been used in experiments designed to induce antitumour immunity with laser–dye interactions (Chen *et al*, 1999). Mice are the ideal model in which to employ GFP-tumours because they are small animals suitable for placement in cameras with restricted space and the tumours generally are relatively near the surface due to the small total body volume of a mouse (Hoffman, 2002b). We found liver metastases easily by GFP imaging, while traditional examination of organs by histopathology is tedious and labour intensive. Twentyman reported (Twentyman *et al*, 1980) an ‘occasional lung metastasis’ from wild-type RIF-1 tumours but did not find liver metastases. However, when GFP-expressing mouse tumours are used in syngeneic immunocompetent mice it is important to consider the potential immunogenicity of GFP. Our findings that PDT mediated by BPD produces a 100% cure rate of RIF-1 tumours that stably express EGFP while having only a small temporary effect against wild-type RIF-1 tumours, suggests that the presence of the ‘foreign’ protein EGFP is recognised by the mouse immune system. However, the observation that RIF-1 EGFP tumours have the same growth rate in C3H mice as wild-type RIF-1 tumours suggests that another immunopotentiating stimulus is necessary to engender the full immune response against the tumour. In our case, this second immune activating stimulus is BPD-mediated PDT. Similar induction of anti-tumour immunity has been reported using a selective photothermal interaction in combination with an intratumour administration of immunoadjuvant (Chen *et al*, 2002). The result of the EGFP expression seems to have been to increase the immunogenicity of the tumour in a controlled manner; that is enough for PDT to produce a curative effect, but not enough to affect growth in control mice. The fact that RIF-1 EGFP can be metastatic emphasises the necessity of the involvement of the host immune system in producing lasting cures by PDT. Even if the local tumour were completely destroyed by PDT, mice may still die of distant metastases.

There have been several reports on the generation of anti-tumour immunity in mouse or rat cancer models by PDT (Korbelik and Cecic, 1999; Korbelik and Dougherty, 1999) and also by a related photothermal technology (Chen *et al*, 2003). It is thought that tumour-specific cytotoxic T lymphocytes can be generated by these therapies and that these account for the long-term memory immune response against the tumour (Korbelik *et al*, 2001). One advantage of using EGFP-expressing tumours to demonstrate PDT-induced antitumour immunity is that it appears that EGFP represents a defined model tumour antigen that can mediate the immune response against the tumour.

The potential immunogenicity of GFP in mice was first reported by Stripecke *et al* (1999) who found that stable expression of EGFP in two mouse tumours inhibited tumour growth and led to formation of cytotoxic CD8 T cells that recognise EGFP-expressing cells. The identification of EGFP 200–208 as an H-2Kd-restricted cytotoxic-T-lymphocyte (CTL) epitope by Gambotto *et al* (2000) further defined EGFP as a model tumour antigen. Other workers have found that implantation of EGFP-expressing tumours in mice has induced the presence of anti-EGFP IgG antibodies in the serum (Brown *et al*, 2001; Spiotto *et al*, 2003).

Cellular and humoral immune response against GFP have been observed in rhesus macaques that underwent haematopoietic stem cell transplantation with EGFP-transduced CD34+ cells; in this study they found that the EGFP-specific CTL responses were MHC-restricted, mediated by CD8+ lymphocytes, and directed against multiple epitopes (Rosenzweig *et al*, 2001). Another study showed that GFP, delivered into dendritic cells (DCs) by an adenoviral vector or following protein pulsing, could significantly modify DC phenotype, into a terminal maturation-like process which enhanced their immunostimulatory capacity (Re *et al*, 2004).

Other foreign antigens have been genetically engineered to be expressed in tumour cells and have been used to increase the humoral and/or cellular immunity against cancer in mice. Ovalbumin (a chicken protein) is processed and presented through MHC I with generation of tumour-specific CTL. This system has been used to test several approaches involving cancer vaccination strategies (Nelson *et al*, 2001; Chung *et al*, 2004; van Broekhoven *et al*, 2004).

It has been shown that tumour cells expressing beta-galactosidase (a bacterial protein) also can induce a strong cellular immune response against the antigen (Wang *et al*, 1995b). (Wang *et al* (1995a) reported that CT26 mouse colon cancer cells expressing beta-galactosidase, formed tumours that could be treated with virus-mediated immunotherapy while the wild-type tumours were resistant.

The fact that we observed a small number of cures (17%) when tumours formed from RIF-1 cells that had been transduced with the empty retroviral vector as a control (RIF-1 EPV, Figure 6) were treated with PDT, and the tumours that recurred (83%) grew significantly slower than recurrent wild-type RIF-1 tumours, shows that not only EGFP can act as a foreign antigen in the present system. Presumably some of the genes in the retrovirus encode proteins that can also act as model tumour antigens, although to a much lesser extent than EGFP.

The generation of a long-term memory immune response as demonstrated by the resistance of cured mice to rechallenge with RIF-1 EGFP cells was not unexpected. Reports have demonstrated that the production of anti-tumour immunity by curing tumours in mice either by PDT (Canti *et al*, 1994) or by other methods (Suzuki *et al*, 2003), and have shown a resistance to rechallenge by the same tumour cells that formed the primary tumour. The baseline level of immunogenicity of the EGFP expressing tumours is confirmed by the data that a rechallenge of mice whose RIF-1 EGFP tumours had been surgically removed with the same RIF-1 EGFP cells led to a significantly reduced rate of tumour growth but no outright rejection. However, the finding that wild-type RIF-1 tumours grew significantly slower in mice cured from RIF-1 EGFP tumours than in naïve mice was surprising. The RIF-1 cell line has always been considered to be poorly immunogenic, but our data implies that it does contain partly effective tumour antigens that can be recognised by the immune system when subjected to stimulation by the PDT-induced creation of anti-tumour immunity.

There is a considerable amount of work remaining to be done to understand these results. Our future work will be focused on the identification of the immune mechanisms behind the immunity generated by PDT against GFP and wild-type tumours. We need to do additional work to establish the role that EGFP is playing in comparison with the retroviral vector used to transduce the cells. However, the presence of anti-GFP antibodies before PDT suggests that the effect is primarily mediated by the foreign jellyfish antigen. We will investigate whether mice cured of RIF-1 EGFP tumours also have immunity against other EGFP-expressing tumours cells syngeneic to C3H mice.
